# Nursing, midwifery, and allied health professions research capacities and cultures: a survey of staff within a university and acute healthcare organisation

**DOI:** 10.1186/s12913-023-09612-3

**Published:** 2023-06-16

**Authors:** S. Palmer, J. Coad, J. Gamble, C. Jones, L. Lees-Deutsch, D. McWilliams, E. Murphy, R. Kneafsey

**Affiliations:** 1 School of Healthcare Sciences, Cardiff University, Cardiff, UK; 2 Centre for Care Excellence, Coventry University and University Hospitals Coventry and Warwickshire NHS Trust, Richard Crossman Building, Jordan Well, Coventry, CV1 5RW UK; 3 School of Health Sciences, Nottingham University Queen’s Medical Centre, Nottingham, UK

**Keywords:** Research culture, Research capacity, Nursing, Midwifery, Allied health professions, Survey

## Abstract

**Background:**

There is an increasing focus on the development of research capacity and culture in Nursing, Midwifery and Allied Health Professions (NMAHP). However, better understanding of the existing research success and skills, motivators, barriers, and development needs of NMAHP professionals is required to inform this development. This study sought to identify such factors within a university and an acute healthcare organisation.

**Methods:**

An online survey, incorporating the Research Capacity and Culture tool, was administered to NMAHP professionals and students at a university and an acute healthcare organisation in the United Kingdom. Ratings of success/skill levels of teams and individuals were compared between professional groups using Mann–Whitney U tests. Motivators, barriers, and development needs were reported using descriptive statistics. Descriptive thematic analysis was used for open-ended text responses.

**Results:**

A total of 416 responses were received (N&M *n* = 223, AHP *n* = 133, Other *n* = 60). N&M respondents were more positive than their AHP counterparts about the success/skill levels of their teams. There were no significant differences between N&M and AHP in their ratings of individual successes/skills. Finding and critically reviewing relevant literature were identified as specific individual strengths; with weaknesses in securing research funding, submitting ethics applications, writing for publication, and advising less experienced researchers. The main motivators for research were to develop skills, increased job satisfaction, and career advancement; whilst barriers included lack of time for research and other work roles taking priority. Key support needs identified included mentorship (for teams and individuals) and in-service training. Open-ended questions generated main themes of ‘Employment & staffing’, ‘Professional services support’, ‘Clinical & academic management’, ‘Training & development’, ‘Partnerships’ and ‘Operating principles’. Two cross-cutting themes described issues common to multiple main themes: ‘Adequate working time for research’ and ‘Participating in research as an individual learning journey’.

**Conclusions:**

Rich information was generated to inform the development of strategies to enhance research capacity and culture in NMAHP. Much of this can be generic but some nuances may be required to address some specific differences between professional groups, particularly related to perceived team success/skills and priorities identified for support and development.

**Supplementary Information:**

The online version contains supplementary material available at 10.1186/s12913-023-09612-3.

## Background

This research sought to understand issues associated with research capacity and culture in Nursing, Midwifery and Allied Health Professions (NMAHP) across two organisations (a university and an acute healthcare organisation). A review of evidence has demonstrated that engagement in research by individual clinicians and healthcare organisations improves performance, including health outcomes and processes of care [1]. Data has continued to demonstrate that research-active organisations perform better on outcomes such as reduced patient mortality [2, 3]; increased colorectal cancer survival [4]; improved ratings of organisational performance [3]; and improved patient experience (incorporating observed teamwork, quality of information received and confidence in doctors) [5]. Staff who work in research-active organisations are also more likely to recommend their organisation as a good place to work or to be treated [5]. Evidence for the benefits of a positive research culture therefore continues to grow.

Development of research capacity and culture-has thus become an important strategic priority in the United Kingdom (UK) and elsewhere. There has been a particular focus on professions outside of Medicine, as these represent most of the health and social care workforce. Indeed, the potential positive impacts of doctoral-educated Nurses working in clinical settings has been supported by a recent scoping review [6]. Within the UK, published research strategies for Nurses [7], Midwives [8] and Allied Health Professionals (AHPs) [9] clearly identify ambitions to transform the culture around research and evidence-based practice. In England, this ambition is supported by the National Institute for Health and Care Research and Health Education England, who provide funding to support clinical academic careers at pre-doctoral, doctoral and post-doctoral levels. These schemes are open to NMAHPs and a wide range of other health and social care professions and require active partnerships between universities and health and social care organisations to support awardees [10]. A recent review of these schemes [11] identified that fewer applications were received from AHPs (14% of applications) and Nurses & Midwives (11.5%) than Medics (37%), although the overall rate of success was similar across professions. The only exception to this was for late postdoctoral, senior and chair applications from applicants without a previous National Institute for Health and Care Research award. In this subgroup, professional background was significant, with a 24.5% success rate for AHPs and other health professionals, 16% for Medics, and 9.1% for Dentists, Nurses & Midwives, and non-health professionals. Applications from institutions associated with a medical school were 2.61 times more likely to be successful [11]. So, specific strategies may be required to support NMAHPs to make applications, to support senior applications from Nurses & Midwives, and to support applications from a wider range of institutions.

An innovative example of a strategic partnership aiming to support NMAHP research is the ‘Centre for Care Excellence’, established in 2020 as a jointly funded initiative between Coventry University (subsequently referred to as ‘university’) and University Hospitals Coventry and Warwickshire NHS Trust (subsequently referred to as ‘healthcare organisation’), West Midlands, UK. The aims of the Centre are to integrate research, innovation, practice development and education to make a positive difference to patient experiences and outcomes and to support clinical and academic staff in health service research, delivery, and education. This partnership helps to address the key principles and obligations of those responsible for supporting clinical academics set by the National Institute for Health and Care Research [10]. It addresses areas highlighted by Henshall and colleagues regarding funding, time, infrastructure, leadership, and partnership [12]. The Centre for Care Excellence has appointed five senior NMAHP clinical academics to help achieve those aims. To inform the development of a strategy to meet the needs of staff in both organisations, an online survey was undertaken to identify existing NMAHP research expertise; determine potential barriers and motivators to research; and identify priorities for staff support and development. This research provides unique insight across a university and acute care healthcare organisation.

The Research Capacity and Culture (RCC) Tool [13] has been successfully used in previous healthcare staff evaluations to identify research skills and successes at organisational, team and individual levels; and personal barriers and motivators for research [14–17]. The present study therefore used the RCC and additional survey questions to address the following aims: 1. To identify the current levels of NMAHP research success and research skills at the team and individual level; 2. To identify the barriers and motivators to NMAHP research; and 3. To identify preferences for support that could be provided by the Centre for Care Excellence.

## Methods

The protocol was approved by the university Faculty of Health & Life Sciences Ethics Committee (Reference P124919) and ratified by the healthcare organisation’s Research & Development Department (Reference GF0441) in line with the harmonised edition of the ‘Governance Arrangements for Research Ethics Committees’ [18]. All methods were carried out in accordance with the Declaration of Helsinki. An anonymous online questionnaire was developed and administered via Joint Information Systems Committee Online Surveys. Embedded within the questionnaire was full study information, a privacy notice and explicit informed consent. Consent was required before progressing to the main questionnaire. Participants were free to exit the questionnaire at any point by exiting and closing their browser.

NMAHP clinicians, academics and students were recruited via email advertisement containing a brief overview of the project and a link to the online questionnaire. Details were also posted in the healthcare organisation’s staff newsletter. Inclusion criteria were: 1. Current undergraduate, postgraduate or doctorate student or staff member at the university or healthcare organisation; 2. Identifies as one of the NMAHP professional titles regulated by the Health & Care Professions Council or the Nursing & Midwifery Council in the UK; 3. Able to provide informed consent; 4. Able to complete the online questionnaire in English; 5. Aged 18 + years. There were no specific exclusion criteria.

The email was sent to all staff within the School of Nursing, Midwifery and Health at the university. NMAHP curriculum leads at the university were asked to forward details to undergraduate and postgraduate students. The Associate Directors for Nursing, Midwifery, and Allied Health Professionals at the healthcare organisation distributed the email to all staff within their practice areas. Finally, the Research and Development Department at the healthcare organisation distributed the email to everybody on their email contact list. A reminder email was sent out two weeks later. To maximise returns an amendment to ethical approval was secured to allow one further round of emails (and associated two-week reminder).

The questionnaire incorporated the following components of the RCC Tool [13]: ‘team’ success or skill level, ‘individual’ success or skill level, ‘barriers’ and ‘motivators’. The RCC tool was selected as it is well-validated and has been used in other NMAHP studies in the UK and Australia, allowing the results to be directly compared. A modified version of the RCC [19] was used in the present study, which employed a 1–9 scale (plus an ‘unsure’ option) for the success or skill levels (1 = no success/skill and 9 = highest possible success/skill). It was decided to exclude the ‘organisational’ success or skill level of the RCC as the Centre for Care Excellence crosses two organisations (university and healthcare organisation) and the items are identical to the ‘team’ component. The following clarification was added before the ‘team’ questions: *“For these questions, please interpret ‘team’ as the group of professionals that you work most closely with in your current role”*. The ‘individual’ questions were prefaced with the following clarification: *“For these questions, please think about your own personal success or skill levels”*. The wording of individual RCC components was as reported by Raschke [19].

Also included were questions about professional background, demographics, professional role, qualifications and the types of support that participants would like to receive from the Centre for Care Excellence (the perceived helpfulness of different types of support, rated from 0 = ‘not at all helpful’ to 4 = ‘extremely helpful’; and the perceived interest in learning more about specific topics, rated from 0 = ‘not at all interested’ to 4 = ‘extremely interested’). These latter questions were developed and refined by the research team based on their professional and research experience. The online questionnaire was piloted by research team members and amended before distribution to participants. The survey opened on 15^th^ July 2021 and the final submission was on 29^th^ October 2021.

### Data analysis

Response rates for the healthcare organisation were estimated based on known staff numbers for N&M (*n* = 2,567) and AHPs (*n* = 1,051). It should be noted that the AHP staff figure included all healthcare scientists (*n* = 536), many of whom are not regulated by the Health & Care Professions Council. Response rates for the university were based on the number of staff on the School of Nursing, Midwifery & Health email distribution list (*n* = 272), although it should be noted that this figure includes support staff and non-NMAHP academic and research staff. For this reason, all N&M, AHP and Other respondents were combined as the numerator for the university. The student response rate was not calculated as, due to non-response, it was impossible to verify which curriculum leads had distributed study details to which NMAHP student groups.

Quantitative data were analysed descriptively, using proportions of response categories, mean and median values as appropriate. Where the numbers of individuals within sub-categories were small (< 5 participants), that information was not reported separately to preserve anonymity. Data for the ‘team’ and ‘individual’ success or skill level were rescaled to a score out of 10 [19] to facilitate comparison with other literature (raw score out of 9 × 10/9). ‘Unsure’ responses were not scored and were excluded from analysis [19] to prevent inappropriate interpretation of what individuals might have meant when they selected this option. Cut-off scores of < 4 (‘low’), ≥ 4 and ≤ 6.9 (‘moderate’), and ≥ 7 (‘high’) were used to categorise success or skill levels [16]. As the Centre for Care Excellence crosses two organisations, the focus of the analysis was by professional group. Additional analyses (such as by organisation or multivariate analysis) were excluded a priori to prevent over-analysis and the potential for type I errors. Due to the relatively low number of Midwives responding, they were combined with Nursing for the purposes of analysis (‘N&M’). Similarly, all AHP groups have been combined (‘AHPs’). Finally, many responses were received from individuals who did not identify as NMAHPs, despite the eligibility criteria stated in recruitment materials. Rather than excluding these data, these have been reported separately as ‘Other’. Differences between N&M and AHP ratings of success or skills levels were explored using non-parametric Mann–Whitney U tests. Bonferroni correction for multiple testing was used to adjust the α level.

Three open-ended questions were posed as part of the questionnaire, as follows: *“Please provide details of any other types of support that you think it would be helpful for the CfCE* [Centre for Care Excellence] *to provide”; “Please provide details of any other topics that you would be interested in learning more about”;* and *“Please use this space to add any further details about the issues addressed in this questionnaire”*. It was clear that respondents did not make a clear distinction between these fields and there was obvious repetition in some instances. A pragmatic decision was therefore made to treat all free text responses as a single data set. Data were exported into a Microsoft Excel spreadsheet which was used to organise the data. Free text responses were analysed using descriptive thematic analysis [20]. Coding was conducted by the lead author (SP) through an iterative process of reading and re-reading the typed comments. The coding list was then used to generate descriptive themes, with accompanying descriptions and illustrative quotations. Codes and the emergent themes and subthemes were discussed in detail and verified by a second researcher (LL-D). Additional cross-cutting themes were developed through discussion and consensus to represent data that was common to multiple main themes.

## Results

A total of *n* = 416 responses were received. Just over half of all respondents (53.6%) were N&M (223/416), AHPs accounted for 32.0% (133/416) and the remainder described a wide range of other roles (60/416, 14.4%) (see Table 1).

**Table 1:** Professional background of respondents. ‘Other’ included a very wide range of self-described roles, including but not limited to health care assistants, medical doctors, pharmacists, managers, researchers, public health practitioners, and staff working in research and development and knowledge services roles

*“Please describe the profession that best describes your current role”*	Role	Number of respondents (%)
**N&M (*n* = 223)**	Registered Nurse	212 (51.0%)
	Midwife	11 (2.6%)
**AHPs (*n* = 133)**	Arts Therapist	0 (0.0%)
	Biomedical Scientist	1 (0.2%)
	Chiropodist/Podiatrist	0 (0.0%)
	Clinical Scientist	5 (1.2%)
	Dietician	19 (4.6%)
	Hearing Aid Dispenser	0 (0.0%)
	Occupational Therapist	28 (6.7%)
	Operating Department Practitioner	13 (3.1%)
	Orthoptist	5 (1.2%)
	Paramedic	6 (1.4%)
	Physiotherapist	30 (7.2%)
	Practitioner Psychologist	3 (0.7%)
	Prosthetist/Orthotist	0 (0.0%)
	Radiographer	18 (4.3%)
	Speech & Language Therapist	5 (1.2%)
**Other (*n* = 60)**	Other	60 (14.4%)
**Total**		**416 (100%)**

*AHPs* Allied Health Professions, *N&M* Nursing & Midwifery

Table 2 below illustrates that most respondents stated that they were healthcare organisation employees (300/416, 72.1%), had > 10 years’ experience working in health (277/416, 75.1%), were female (343/416, 82.5%) and were aged ≥ 40 years (267/416, 64.2%). Almost half reported that they had postgraduate qualifications (197/416, 47.4%), with 8.7% having a doctorate (36/416). 30.5% (127/416) identified their primary role as a ‘clinician’, with a very wide range of other roles identified. The response rate was 7.2% (184/2567) for N&M and 7.6% for AHPs (80/1051) employed by the healthcare organisation. The response rate was 25.4% (69/272) for staff employed by the university.

**Table 2:** Information about demographics, professional role, and qualifications. Fuller details are provided in Additional file 1

	**N&M** **(*n* = 223)**	**AHPs** **(*n* = 133)**	**Other** **(*n* = 60)**	**Total** **(*n* = 416)**
***“I am completing this questionnaire as a…”***				
Student	6 (2.7%)	25 (18.8%)	16 (26.7%)	**47 (11.3%)**
Employee of Coventry University	33 (14.8%)	28 (21.1%)	8 (13.3%)	**69 (16.6%)**
Employee of University Hospitals Coventry & Warwickshire NHS Trust	184 (82.5%)	80 (60.3%)	36 (60%)	**300 (72.1%)**
***“How long have you been working in the health industry?”***				
< 5 years	7 (3.2%)	16 (14.8%)	8 (18.2%)	**37 (10.0%)**
5–10 years	42 (19.4%)	17 (15.7%)	5 (11.4%)	**55 (14.9%)**
> 10 years	168 (77.4%)	75 (69.4%)	31 (70.5%)	**277 (75.1%)**
***“What is your age?”***				
< 20	0 (0.0%)	5 (3.8%)	0 (0.0%)	**5 (1.2%)**
20–29	11 (4.9%)	16 (12.0%)	13 (21.7%)	**40 (9.6%)**
30–39	52 (23.3%)	41 (30.8%)	11 (18.3%)	**104 (25.0%)**
40–49	75 (33.6%)	41 (30.8%)	15 (25.0%)	**131 (31.5%)**
50–60	74 (33.2%)	29 (21.8%)	16 (26.7%)	**119 (28.6%)**
> 60	11 (4.9%)	1 (0.8%)	5 (8.3%)	**17 (4.1%)**
***“What is your gender?”***				
Male	25 (11.2%)	21 (15.8%)	20 (33.3%)	**66 (15.9%)**
Female	195 (87.4%)	109 (82.0%)	39 (65.0%)	**343 (82.5%)**
Prefer not to say	3 (1.3%)	2 (1.5%)	1 (1.7%)	**6 (1.4%)**
Other	0 (0.0%)	1 (0.8%)	0 (0.0%)	**1 (0.2%)**
***“My primary role is…”***				
Clinician	56 (25.1%)	57 (42.9%)	14 (23.3%)	**127 (30.5%)**
Lecturer	23 (10.3%)	19 (14.3%)	1 (1.7%)	**43 (10.3%)**
Manager	36 (16.1%)	13 (9.8%)	10 (16.7%)	**59 (14.2%)**
Practice educator	16 (7.2%)	6 (4.5%)	1 (1.7%)	**23 (5.5%)**
Researcher	7 (3.1%)	3 (2.3%)	7 (11.7%)	**17 (4.1%)**
Other	54 (24.2%)	9 (6.8%)	10 (16.7%)	**73 (17.5%)**
***“Currently, my highest qualification is…”***				
Doctorate	10 (4.5%)	14 (10.5%)	12 (20.0%)	**36 (8.7%)**
Other higher degree e.g. MRes, MSc	55 (24.7%)	41 (30.8%)	20 (33.3%)	**116 (27.9%)**
PGCE	2 (0.9%)	5 (3.8%)	0 (0.0%)	**7 (1.7%)**
Other postgraduate qualification (including professional)	21 (9.4%)	13 (9.8%)	4 (6.7%)	**38 (9.1%)**

*AHPs* Allied Health Professions, *MRes* Master of Research, *MSc* Master of Sciences, *N&M* Nursing & Midwifery, *PGCE* Postgraduate Certificate in Education

Tables 3, 4, 5, 6 report separate elements of the RCC tool. Table 3 relates to the success or skill level of participants’ teams. N&M respondents rated ‘does planning that is guided by evidence’ and ‘supports a multi-disciplinary approach to research’ as ‘high’, whilst AHPs failed to rate any items as ‘high’. Both groups rated ‘has applied for external funding as ‘low’. Overall, AHPs were more negative than N&M respondents about the skill or success level of their teams. There were statistically significant differences between the ratings of N&M and AHP respondents for 13 of the 19 items and in all cases AHPs rated the items lower. The ‘Other’ group reported higher median values on almost all individual items, with the exception of ‘has incentives & support for mentoring activities’ which was rated the same as N&M.

**Table 3:** Median (IQR) ratings for the success or skill level of the team

*“Please rate your team’s current success or skill level for each of the following aspects”*							
	**Other *n* = 60**	**N&M *n* = 223**	**N&M Summary Level**	**AHPs *n* = 133**	**AHP Summary Level**	*p*-value N&M versus AHP	**‘Unsure’ n (%)**
Has adequate resources to support staff research training	7.78 (3.33)	6.67 (3.34)	Moderate	4.44 (4.45)	Moderate	*p < 0.001	45 (10.8)
Has funds, equipment or admin to support research activities	7.78 (5.56)	5.56 (4.73)	Moderate	3.33 (5.01)	Low	*p < 0.001	66 (15.9)
Does team level planning for research development	7.78 (5.56)	5.56 (5.56)	Moderate	3.33 (4.73)	Low	*p < 0.001	57 (13.7)
Ensures staff involvement in developing that plan	7.78 (4.45)	5.56 (5.56)	Moderate	3.33 (5.56)	Low	*p = 0.001	47 (11.3)
Has team leaders that support research	8.89 (2.22)	6.67 (4.45)	Moderate	5.56 (5.56)	Moderate	NS *p* = 0.007	46 (11.1)
Provides opportunities to get involved in research	7.78 (3.05)	6.67 (4.45)	Moderate	4.44 (4.45)	Moderate	*p = 0.001	35 (8.4)
Does planning that is guided by evidence	8.89 (3.33)	7.78 (3.33)	High	6.67 (5.56)	Moderate	*p < 0.001	45 (10.8)
Has consumer involvement in research activities/planning	7.78 (4.45)	5.56 (4.45)	Moderate	3.33 (5.56)	Low	*p < 0.001	79 (19.0)
Has applied for external funding for research	7.23 (7.78)	3.33 (5.56)	Low	3.33 (6.67)	Low	NS *p* = 0.604	109 (26.2)
Conducts research activities relevant to practice	7.78 (3.33)	6.67 (5.56)	Moderate	4.44 (5.56)	Moderate	NS *p* = 0.017	50 (12.0)
Supports applications for research scholarships/ degrees	8.89 (5.28)	5.56 (6.67)	Moderate	5.56 (7.78)	Moderate	NS *p* = 0.511	80 (19.2)
Has mechanisms to monitor research quality	7.78 (4.44)	5.56 (6.67)	Moderate	3.33 (5.56)	Low	*p = 0.002	79 (19.0)
Has identified experts accessible for research advice	7.78 (3.89)	6.67 (5.56)	Moderate	5.56 (6.12)	Moderate	NS *p* = 0.018	74 (17.8)
Disseminates research results at research forums/seminars	7.78 (3.33)	6.67 (5.56)	Moderate	4.44 (6.67)	Moderate	NS *p* = 0.036	63 (15.1)
Supports a multi-disciplinary approach to research	8.89 (3.33)	7.78 (4.45)	High	5.56 (5.56)	Moderate	*p = 0.001	52 (12.5)
Has incentives & support for mentoring activities	6.67 (4.45)	6.67 (5.56)	Moderate	3.33 (5.56)	Low	*p < 0.001	59 (14.2)
Has external partners (e.g. universities) engaged in research	7.78 (2.50)	6.67 (5.56)	Moderate	3.33 (6.67)	Low	*p < 0.001	80 (19.2)
Supports peer-reviewed publication of research	8.89 (2.22)	6.67 (5.56)	Moderate	4.44 (6.67)	Moderate	*p = 0.001	66 (15.9)
Has software available to support research activities	7.78 (6.39)	5.56 (6.67)	Moderate	2.78 (5.56)	Low	*p < 0.001	82 (19.7)
**Median (IQR)**	**7.78 (2.78)**	**6.67 (4.45)**	**Moderate**	**4.44 (5.84)**	**Moderate**	*p = 0.001	N/A

Bonferroni correction applied (α = 0.05 ÷ 19 = *p* < 0.003). *Statistically significant difference N&M versus AHPs (Mann–Whitney U test, *p* < 0.003)

*NS* Not significant, *AHPs* Allied Health Professions, *N&M* Nursing & Midwifery

Table 4 reports data about the individual success/skill levels of respondents, demonstrating consistency across both N&M and AHP respondents, with no statistically significant differences in ratings between groups. The top-rated items related to ‘finding relevant literature’ and ‘critically reviewing the literature’, both being rated as ‘high’ by both groups. Perceived individual success/skill levels were ‘low’ for ‘securing research funding’, ‘submitting an ethics application’, ‘writing for publication in peer-reviewed journals’ and ‘providing advice to less experienced researchers’. The ‘Other’ group reported higher median scores on all items, except for ‘critically reviewing the literature’ where the median value was equal to the other groups.

**Table 4:** Median (IQR) ratings for the success or skill level of the individual respondents

***“Please rate your own current success or skill level for each of the following aspects”***							
	**Other** ***n* = 60**	**N&M** ***n* = 223**	**N&M Summary Level**	**AHPs** ***n* = 133**	**AHP Summary Level**	*p*-value N&M versus AHP	**‘Unsure’** **n (%)**
Finding relevant literature	8.89 (2.22)	7.78 (3.33)	High	7.78 (3.33)	High	**NS *p* = 0.944**	5 (1.2)
Critically reviewing the literature	7.78 (2.22)	7.78 (3.33)	High	7.78 (4.45)	High	**NS *p* = 0.777**	11 (2.6)
Using a computer referencing system (e.g. Endnote)	7.78 (4.44)	6.67 (5.56)	Moderate	6.67 (4.45)	Moderate	**NS *p* = 0.517**	18 (4.3)
Writing a research protocol	7.78 (3.33)	4.44 (5.84)	Moderate	5.56 (4.73)	Moderate	**NS *p* = 0.077**	17 (4.1)
Securing research funding	5.56 (5.56)	2.22 (4.45)	Low	2.22 (3.33)	Low	**NS *p* = 0.643**	50 (12.0)
Submitting an ethics application	6.67 (5.56)	3.33 (5.56)	Low	3.33 (6.67)	Low	**NS *p* = 0.293**	40 (9.6)
Designing questionnaires	7.78 (3.33)	5.56 (5.56)	Moderate	5.56 (4.45)	Moderate	**NS *p* = 0.951**	23 (5.5)
Collecting data (e.g. surveys, interviews)	7.78 (4.44)	6.67 (4.45)	Moderate	6.67 (4.45)	Moderate	**NS *p* = 0.589**	21 (5.0)
Using computer data management systems	7.23 (5.01)	5.56 (5.56)	Moderate	5.56 (5.56)	Moderate	**NS *p* = 0.984**	26 (6.3)
Analysing qualitative research data	7.78 (5.56)	5.56 (5.56)	Moderate	4.44 (5.56)	Moderate	**NS *p* = 0.959**	21 (5.0)
Analysing quantitative research data	7.78 (4.45)	4.44 (4.45)	Moderate	4.44 (5.56)	Moderate	**NS *p* = 0.385**	19 (4.6)
Writing a research report	8.34 (4.44)	4.44 (6.67)	Moderate	5.56 (5.56)	Moderate	**NS *p* = 0.113**	19 (4.6)
Writing for publication in peer-reviewed journals	7.78 (4.73)	3.33 (5.56)	Low	3.33 (5.56)	Low	**NS *p* = 0.624**	27 (6.5)
Providing advice to less experienced researchers	6.67 (5.56)	3.33 (5.56)	Low	3.33 (5.56)	Low	**NS *p* = 0.903**	24 (5.8)
**Median (IQR)**	**7.78** **(3.33)**	**5.56** **(5.56)**	**Moderate**	**5.56** **(5.01)**	**Moderate**	**NS *p* = 0.532**	N/A

Bonferroni correction applied (α = 0.05 ÷ 14 = *p* < 0.004)

*AHPs* Allied Health Professions, *N&M* Nursing & Midwifery, *N/A* Not Applicable, *NS* Not significant

The major personal barriers to research selected by participants were ‘lack of time for research’ and ‘other work roles take priority’, where a majority of all respondents selected these items (Table 5). There was general consistency between professional groups in the rank order of items, perhaps apart from ‘lack of a coordinated approach to research’ which was ranked 7^th^ by AHPs (selected by 41.4% of respondents) compared to 14^th^ by N&M (18.4% of respondents). AHPs also tended to identify more barriers than N&M, with 13 of the 18 items selected by a higher proportion of AHPs than N&M.

**Table 5:** Personal barriers to research [rank] (%)

*“What are the barriers to research for you personally? Tick as many as apply.”*	Other (*n* = 60)	N&M (*n* = 223)	AHPs (*n* = 133)	Total (*n* = 416)
Lack of time for research	[1] 44 (73.3%)	[2] 174 (78.0%)	[1] 111 (83.5%)	[1]** 329** **(79.1%)**
Other work roles take priority	[2] 41 (68.3%)	[1] 175 (78.5%)	[2] 107 (80.5%)	[2]** 323** **(77.6%)**
Lack of suitable backfill	[= 5] 20 (33.3%)	[4] 104 (46.6%)	[3] 77 (57.9%)	[3]** 201** **(48.3%)**
Desire for work/life balance	[7] 19 (31.7%)	[3] 107 (48.0%)	[6] 61 (45.9%)	[4]** 187** **(45.0%)**
Lack of funds for research	[3] 26 (43.3%)	[= 5] 91 (40.8%)	[4] 68 (51.1%)	[5]** 185** **(44.5%)**
Lack of administrative support	[4] 21 (35.0%)	[7] 76 (34.1%)	[5] 63 (47.4%)	[6]** 160** **(38.5%)**
Lack of skills for research	[12] 15 (25.0%)	[= 5] 91 (40.8%)	[8] 49 (36.8%)	[7]** 155** **(37.3%)**
Lack of support from management	[= 8] 17 (28.3%)	[8] 71 (31.8%)	[= 9] 46 (34.6%)	[8]** 134** **(32.2%)**
Lack of software for research	[= 5] 20 (33.3%)	[= 10] 58 (26.0%)	[11] 45 (33.8%)	[9]** 123** **(29.6%)**
Lack access to equipment for research	[= 8] 17 (28.3%)	[12] 55 (24.7%)	[= 9] 46 (34.6%)	[10]** 118** **(28.4%)**
Other personal commitments	[14] 10 (16.7%)	[9] 67 (30.0%)	[13] 35 (26.3%)	**[= 11] 112** **(26.9%)**
Lack of a coordinated approach to research	[= 10] 16 (26.7%)	[14] 41 (18.4%)	[7] 55 (41.4%)	**[= 11] 112** **(26.9%)**
Intimidated by fear of getting it wrong	[= 10] 16 (26.7%)	[13] 49 (22.0%)	[12] 38 (28.6%)	[13]** 103** **(24.8%)**
Intimidated by research language	[15] 9 (15.0%)	[= 10] 58 (26.0%)	[14] 32 (24.1%)	[14]** 99** **(23.8%)**
Not interested in research	[17] 3 (5.0%)	[15] 34 (15.2%)	[16] 17 (12.8%)	[15]** 54** **(13.0%)**
Isolation	[13] 11 (18.3%)	[16] 16 (7.2%)	[15] 22 (16.5%)	[16]** 49** **(11.8%)**
Lack of library/Internet access	[16] 4 (6.7%)	[17] 9 (4.0%)	[17] 10 (7.5%)	[17]** 23** **(5.5%)**
Other	[18] 2 3.3%	[18] 3 (1.3%)	[18] 5 (3.8%)	[18]** 10** **(2.4%)**

Results are presented in rank order of the total mean scores (final column)

*AHPs* Allied Health Professions, *N&M* Nursing & Midwifery

Table 6 illustrates the personal motivators to do research. Again, there was a lot of consistency between professions in the items identified, with ‘to develop skills’, ‘increased job satisfaction’ and ‘career advancement’ being the top three items selected overall. The only item that varied substantially in terms of rank was ‘links to universities/clinical services’ which was ranked joint 6^th^ by AHPs (chosen by 45.1% of respondents) but only ranked 11^th^ by N&M (27.8% of respondents).

**Table 6:** Personal motivators to do research [rank] (%)

*“What are the motivators to do research for you personally? Tick as many as apply.”*	Other	N&M (*n* = 223)	AHPs (*n* = 133)	Total (*n* = 416)
To develop skills	[1] 44 (73.3%)	[1] 178 (79.8%)	[1] 102 (76.7%)	[1]** 324** **(77.9%)**
Increased job satisfaction	[2] 39 (65.0%)	[2] 135 (60.5%)	[2] 90 (67.7%)	[2]** 264** **(63.5%)**
Career advancement	[4] 33 (55.0%)	[3] 115 (51.6%)	[3] 79 (59.4%)	[3]** 227** **(54.6%)**
Problem identified that needs changing	[= 8] 26 (43.3%)	[4] 99 (44.4%)	[4] 69 (51.9%)	[4]** 194** **(46.6%)**
To keep the brain stimulated	[3] 37 (61.7%)	[5] 93 (41.7%)	[8] 59 (44.4%)	[5]** 189** **(45.4%)**
Dedicated time for research	[= 8] 26 (43.3%)	[6] 91 (40.8%)	[= 6] 60 (45.1%)	[6]** 177** **(42.5%)**
Increased credibility	[6] 29 (48.3%)	[7] 77 (34.5%)	[5] 67 (50.4%)	[7]** 173** **(41.6%)**
Opportunities to participate at own level	[5] 29 (48.3%)	[8] 75 (33.6%)	[10] 53 (39.8%)	[8]** 157** **(37.7%)**
Links to universities/clinical services	[7] 28 (46.7%)	[11] 62 (27.8%)	[= 6] 60 (45.1%)	[9]** 150** **(36.1%)**
Mentors available to supervise	[= 12] 17 (28.3%)	[9] 74 (33.2%)	[9] 58 (43.6%)	[10]** 149** **(35.8%)**
Desire to prove a theory or hunch	[10] 24 (40.0%)	[10] 67 (30.0%)	[11] 50 (37.6%)	[11]** 141** **(33.9%)**
Research written into role description	[11] 21 (35.0%)	[12] 61 (27.4%)	[13] 43 (32.3%)	[12]** 125** **(30.0%)**
Research encouraged by managers	[= 12] 17 (28.3%)	[13] 55 (24.7%)	[12] 47 (35.3%)	[13]** 119** **(28.6%)**
Grant funds	[= 12] 17 (28.3%)	[14] 52 (23.3%)	[14] 42 (31.6%)	[14]** 111** **(26.7%)**
Colleagues doing research	[= 15] 14 (23.3%)	[16] 45 (20.2%)	[15] 37 (27.8%)	[15]** 96** **(23.1%)**
Study or research scholarships available	[= 15] 14 (23.3%)	[15] 49 (22.0%)	[17] 27 (20.3%)	[16]** 90** **(21.6%)**
Forms part of postgraduate study	[17] 11 (18.3%)	[17] 38 (17.0%)	[16] 33 (24.8%)	[17]** 82** **(19.7%)**
Other	[18] 2 (3.3%)	[18] 10 (4.5%)	[18] 9 (6.8%)	[18]** 21** **(5.0%)**

Results are presented in rank order of the total mean scores (final column)

*AHPs* Allied Health Professions, *N&M* Nursing & Midwifery

Data presented in Additional file 2 illustrates that, overall, the top three types of support that people would find helpful were ‘mentorship for my team’, ‘mentorship for me’ and ‘in-service training with my team’. However, none of those items featured in the top 3 ranked items for AHPs, suggesting a difference in the support required by the different professional groups. AHPs ranked ‘support with statistical analysis’, ‘support with writing for publications’ and ‘support with grant applications’ as the top 3 ranked items. Finally, Additional file 3 illustrates that the overall top 3 topics of interest were ‘service evaluation’, ‘funding opportunities’ and ‘audit’, although the latter topic was only ranked 10^th^ by AHPs (N&M ranked this 1^st^), again suggesting some differences between professional groups. The table highlights several other differences in ranked priorities between professional groups.

### Open-ended questions findings

Typed responses were received from 80 of 416 respondents (19.2%). Responses varied in depth and length, but it was possible to undertake a descriptive thematic analysis to generate six separate ‘themes’, which are outlined below with illustrative extracts.

### Theme 1: employment & staffing

This theme captured individuals’ expressed needs related to protected time for research and research training, manageable workloads, backfill for posts, investment in staff and setting up of honorary contracts: *TIME—there is absolutely no time at work to do anything more than the most basic in relating to teaching and learning—no staff—poor [morale]—no point [AHP, university]; Clinician[s] need regular allocated time to undertake research [N&M, healthcare organisation], I feel that as nurses caring for patient[s] we do not have time to do the research [N&M, healthcare organisation];* and *The biggest issue for my team would be backfill…[AHP, healthcare organisation]*. Establishing joint clinical academic posts and integrating research into job roles was seen as particularly important: *… members of staff would like feedback on what is being done to stimulate the production of [permanent] clinical academic posts*… *I want to be on the ground, seeing the problems that clinical staff face head on, at the same time as doing research. Why are posts like this so hard to come by? [AHP, university]*.

### Theme 2: professional services support

Respondents requested help with access to and support from a range of services, including library, information technology and statistical analysis: *Access to Cov Uni Library [N&M, healthcare organisation]; Access to statistician readily—we need to book in advance currently [AHP, healthcare organisation]; and library resources [N&M, healthcare organisation]*. Access to software was highlighted by respondents from both organisations: *Access to software such as SPSS [Other, healthcare organisation]; Links with IT Services re new research software [AHP, university];* and *Access to Endnote, SPSS and relevant softwares that would help in research [AHP, healthcare organisation]*. The potential role of library services in supporting evidence-based practice was well-recognised: *I think the library is best placed to teach critical appraisal and literature searching [AHP, healthcare organisation];* and *Library assistance with literature reviews—both Uni & Practice based libraries [N&M, university].*

### Theme 3: clinical and academic management

This theme articulated the importance of the Centre for Care Excellence working closely with clinical and academic managers to enhance the perceived value of research and perhaps indicates the need for culture shift in some areas: *I believe the most useful support would be to promote and encourage recognition of the value of research at a managerial level. The desire of staff to participate is not enough when managers do not value research and see it as a resource ‘cost’ rather than a resource ‘benefit’ [AHP, Student]*; *To promote the importance of research with clinical managers in order that they plan services to include research activity (N&M, university]*; *management support [N&M, healthcare organisation]*; and *We desperately need more time, more support and better leadership [AHP, university].*

### Theme 4: training & development

There were many ideas for additional training and development, some very specific to individual professions and clinical specialties. This included specific ideas about how training and development should be delivered, such as drop-in sessions, education with specific departments (which might deliver basic information that could then be built upon) and skills identification. There were additional ideas for training on specific research methods, including mixed methods, health economics, data visualisation and infographics, product evaluation and codesign and *innovative approaches to research [AHP, university]*. There were requests for support around dissemination, including identifying opportunities for dissemination and publishing (including self-publishing), and support around funding, such as *Writing grants and how to approach external funding [AHP, healthcare organisation]*. Finally, some wider principles around training and development were articulated, such as learning how to build small bits of work into more substantial studies, ethics, theoretical frameworks, sustainability of research, applying research in teaching, and leadership and mentorship. There was some overlap with the theme of ‘Partnerships’, in that *joint seminars between CU* [Coventry University] *and UHCW* [University Hospitals Coventry & Warwickshire NHS Trust] *[N&M, university]* and *promotion of joint research-related events [Other, university]* were seen as potential benefits*.*

### Theme 5: partnerships

Data relating to partnerships was prevalent throughout and seemed to be perceived as a key benefit of the Centre for Care Excellence. Opportunities for partnerships included those between the university and the healthcare organisation but also externally. For less experienced researchers, building skills as part of a more experienced team was seen as potentially valuable: *opportunities to build up skills by participating in a minor research role i.e. part of team where more experienced others lead & co-ordinate [N&M, university]* and *Knowing what research is going on at UH and the university that perhaps AHPs can get involved in delivering as a first step into research [AHP, healthcare organisation]*. The importance of building teams was highlighted by *Team Approach rather than an individual responsibility [AHP, university];* and *Teams with a vision to achieve specific research aims together [AHP, university].* It was evident that more experienced researchers were very keen to use their skills to support others. Potential practical benefits of partnerships, such as sharing skills and support and waiving of publication fees were identified.

### Theme 6: operating principles

The final theme described some of the hopes and aspirations of respondents about how the Centre for Care Excellence should work in practice. There was a call to be open and welcoming, including sensitive support for less experienced individuals, support for other professions that don’t technically fall under the NMAHP banner, and consideration of accessibility to people working off the main university and healthcare organisation sites (such as the university London campus). The visibility of the Centre for Care Excellence, including a web presence and contact details was identified as being very important. *All above accessible irrespective of seniority and based on individual interest [N&M, healthcare organisation];* and *My hope […] is that CfCE* [Centre for Care Excellence] *will become a non-hierarchical, welcoming/accepting and creative community of practice, that looks towards what can be done, rather than what can't [AHP, university].*

Two rich cross-cutting themes were identified. These described issues common to multiple main themes, as follows:

### Cross-cutting theme 1: adequate working time for research

Respondents described provision of adequate time as a fundamental enabler of research activities. A lack of available time was seen as prohibitive to developing research skills and collaborations, availing of funding, conducting research, and accessing research-related services. This theme was evident across healthcare and university settings. Enabling time release was framed as a managerial responsibility, although respondents were cognisant of service delivery pressures. Time for research at the healthcare organisation was expressed as a pressure within the context of clinical work. Similar time pressures were felt at the university, expressed alongside teaching workloads. Time solutions were proposed in both settings: *funding needed without it we can’t achieve backfill [AHP, healthcare organisation]* and *What we need most is to be released from teaching activity [N&M, university]*.

Participants expressed burden in relation to accomplishing research during working time. Such working time is assigned through timetabling and shift rostering to patient or student facing activity. Finding time within the context of service delivery was ultimately described as *needing time to research within my current role [N&M, university]*. Nevertheless, the issue was not solely finding time, as research was inextricably linked to the need *to integrate research into role [N&M, healthcare organisation]*. Hence, the process of achieving time for research activities must be negotiated and arises from the desire to integrate this within working time and a role which includes research activity. This may require the development of new timetables, shift rosters and job plans. Time, therefore, seems to be a pivotal point of potential negotiation between employees and managers, although the negotiation required may vary across organisations, and between and within departments.

Managers were urged to consider research when planning, for example, *…to promote the importance of research with clinical managers to plan services which include research activity [N&M, university]*. Managerial buy-in was also expressed as a continuum, from research recognition to the allocation of protected time for research, described as *to allocate dedicated time for research activity… [N&M, healthcare organisation]* and *needing to provide protected time… [N&M, healthcare organisation]*. A core message from participants was that managers needed to recognise the benefits of research and support research activity, to avoid respondents having to choose between clinical/educational work and research.

### Cross-cutting theme 2: participating in research as an individual learning journey

This theme embodies the voices of healthcare or university participants who wished to acquire relevant skills to participate in research. For some, this related to fundamental support for literature reviews or statistical analysis, whilst others sought to actively understand how to forge links between the University and Hospital. Some participants expressed barriers to research training, navigating funding and routes to clinical academic posts or career pathways. Those offering solutions did so in terms of accessing courses or sharing their skills.

The need for research skills acquisition was clearly expressed at different levels, from students through to senior staff. Individuals with a desire to participate in research at an exploratory level were able to clearly articulate their needs, such as *…library assistance with literature reviews… [N&M, university]*. For those having previously completed academic or quality improvement work in practice this was expressed as needing *short courses to write up or build on mini projects already started… [Other, healthcare organisation]*. Those wanting to undertake research suggested positive strategies such as *opportunities to engage with existing research to build confidence and skills participating as part of a team with more experienced researchers… [N&M, university]* and *working with discreet tasks and responsibilities would be hugely beneficial [AHP, university]*. However, others felt *it would not be supported by management [AHP, Student]*.

For those participants wishing to develop clinical academic opportunities, ideas were expressed as facilitating dialogue or creating links between the University and Hospital, for example *the promotion of joint research events [Other, university]* and *establishing links with people employed in UHCW* [University Hospitals Coventry & Warwickshire NHS Trust] *and University [Other, university]*. An opportunity for lecturers and clinical staff *to collaborate on a project…* was seen as*… priceless [N&M, university]*. For those staff in the process of doing research, guidance was needed *to understand the myriad of processes involved in gaining grant applications and ethics [AHP, university]*. Conversely, staff with existing research expertise wanted to be linked with *others who have a research interest to share their skills [Other, healthcare organisation]*.

Throughout, participants expressed a lack of support from management, assumed to be line managers, where it was stated explicitly in the context of patient care delivery that *management [are] not supportive of research* and that *PG Study is worthwhile if it develops a clinical skill, not an academic one [AHP, Student]*. From the University perspective this was expressed similarly: *It is the burden of responsibility for teaching that always takes priority and therefore many academics never get the opportunity to be academic [AHP, university]*.

## Discussion

To our knowledge, this is the first NMAHP survey using the RCC tool to explore issues related to research capacity and culture across both university and healthcare settings. We also believe that it is the largest (*n* = 416) such survey conducted to date. This study has demonstrated the research successes and skills, barriers and motivators, and preferences for support in this staff group. A study by Matus and colleagues [16] (*n* = 320) used the RCC to explore the research capacity and culture of AHPs within one Australian healthcare organisation. We included a much wider range of health professionals across two separate organisations. A major benefit of using the RCC tool was that we could directly compare our findings with those from previous literature.

It was interesting that AHPs were generally less positive about their teams’ success or skill levels than their N&M colleagues, although the basis for this difference is unclear. It could indicate a genuine difference between groups, but ratings of perceived success or skills could also be moderated by expectations (i.e. if expectations are high but are not met, then success ratings may be lower and vice versa). The relative expectations of different professional groups are currently unknown but could form an interesting focus for future research. In a previous UK study of Nurses and AHPs (*n* = 224), Luckson and colleagues [15] found no significant differences between groups in their ratings of team success/skill, although ratings were higher in a research-focused hospital (mean 5.28) relative to a non-research-focused hospital (mean 3.61, *p* < 0.001). Although the AHP ratings in our study were similar to those of Luckson and colleagues [15] (median 4.44 versus mean 5.10 respectively), our N&M ratings were much higher (median 6.67 versus mean 4.51 respectively). This may mean that our N&M sample were particularly positive about their teams.

The most highly rated items at the team level in our study, especially by N&M respondents, were ‘does planning that is guided by evidence’ and ‘supports a multi-disciplinary approach to research’. These latter items were rated more positively by N&M colleagues, perhaps due to historical roles as generators of data for research being led by other health professional groups. Cordrey and colleagues [17] identified ‘does planning that is guided by evidence’ and ‘has leaders that support research’ as the top two items rated as team successes/skills by AHPs (*n* = 93) in one UK large healthcare organisation, with ‘supports a multi-disciplinary approach to research’ rated third. Matus and colleagues [16] found little distinction between AHPs’ ratings of different team items, although all three of those items were within the large group of 9 items rated most positively in their study.

In the present investigation, there were no significant differences between N&M and AHP groups when rating their individual success or skills. Luckson and colleagues [15] similarly found no statistically significant differences between AHP and Nursing cohorts in the mean individual success/skill scores (mean 4.54 and 4.24 respectively), although those figures were slightly lower than in our study (N&M and AHP both median 5.56). Luckson and colleagues [15] found that scores were significantly higher in a research-focused hospital (mean 4.6) relative to a non-research-focused hospital (mean 3.87, *p* = 0.003). Ratings for individual success/skill were highest in our study for ‘finding relevant literature’ and ‘critically reviewing the literature’. These two items mirrored the findings for AHP groups in Australia [16] and the UK [17]. Key individual weaknesses identified in the present study for N&M and AHP were ‘securing research funding’, ‘submitting an ethics application’, writing for publication in peer-reviewed journals’ and ‘providing advice to less experienced researchers’. Matus and colleagues [16] identified the same four items as the greatest individual weaknesses in their study. Cordrey and colleagues [17] also identified the same four items in their bottom five, along with ‘writing a research protocol’. This suggests consensus across the available literature in identified individual strengths and weaknesses.

The top two personal barriers to research identified in the present study were ‘lack of time for research’ and ‘other work roles take priority’, both being selected by a majority of respondents in the N&M, AHP and Other groups. These items mirrored the top two barriers identified for Australian and UK AHPs [14, 17]. Pager and colleagues [14] used the RCC tool to explore the motivators and barriers to AHP research capacity in an Australian primary healthcare organisation (*n* = 85). A majority of AHP respondents in our study also selected ‘lack of suitable backfill’ as a barrier but no other items were selected by a majority of the other two professional groups. Backfill may be a particular issue for AHPs as they are more likely to work Monday-Friday daytime working hours, as opposed to N&M colleagues who are more likely to work shift patterns. This lack of flexibility in AHP working patterns may make it more difficult to identify appropriate backfill. A range of other barriers were identified by a majority of participants in previous research. For example, Pager and colleagues [14] identified ‘desire for work/life balance’ (57%), ‘lack of funds for research’ (55%), ‘lack of skills for research’ (54%) and ‘lack of suitable backfill’ (52%); whilst Cordrey and colleagues [17] identified ‘lack of skills for research’ (63%) and ‘lack of suitable backfill’ (54%). This may suggest that our sample were slightly less pessimistic than those in prior studies.

Three items were identified by most respondents in the present survey as motivators to research: ‘to develop skills’ (78%), ‘increased job satisfaction’ (64%) and ‘career advancement’ (55%). These mirrored the top three items identified by Cordrey and colleagues [17] (87%, 72% and 67% respectively) and the top two of these also matched the findings by Pager and colleagues [14] (‘to develop skills’ 81% and ‘increased job satisfaction’ 68%). Three further items were selected by a majority of respondents in the study by Cordrey and colleagues [17]: ‘problem identified that needs changing’ (55%), ‘to keep the brain stimulated’ (53%) and ‘increased credibility’ (53%). Pager and colleagues [14] found that one further item was identified by a majority: ‘problem identified that needs changing’ (53%). Interestingly, some of the ‘motivators’ in the RCC tool might also be considered as ‘enablers’, that is they could act to help professionals to become research active as well as being a product of research activity. The items potentially meeting both descriptors include ‘Dedicated time for research’, ‘Links to universities/clinical services’, ‘Mentors available to supervise’, ‘Research written into role description’, ‘Research encouraged by managers’, ‘Grant funds’ and ‘Study or research scholarships available’. It is not necessarily easy to determine the precise cause-effect relationships between such factors. However, there seems to be a lot of consistency across the literature in the identified motivators.

Our study identified slight nuances between professional groups in the types of support that were rated as helpful. Although mentorship and in-service training were identified as important when all groups were combined, AHPs identified alternative priorities for support. This may mean that a tailored approach to support may be required for different professional groups.

The analysis of the open-ended questions provided some very useful information that contextualised and supported the quantitative data analysis. In particular, respondents provided some excellent suggestions about what would help staff to become more research active. These were clearly articulated within the cross-cutting themes, and included protecting time for research; integrating research into job roles; individualising skills acquisition; building research partnerships; and ensuring management support.

Overall, the current investigation provides information to guide development of a strategy to enhance NMAHP research capacity and culture. A systematic review of model frameworks for research building research capacity for AHPs [21] identified three themes: ‘supporting clinicians in research’, ‘working together’ and ‘valuing research for excellence’. Their overarching capacity-building framework illustrated an interdependence between items, and the need for an integrated approach to implementation that has clear management and leadership support. Avery and colleagues [22] surveyed NMAHP applicants to doctoral and post-doctoral fellowship schemes offered by the National Institute for Health and Care Research. They found that interactions with colleagues in research positions was a key driver to generating interest in research. Unsurprisingly, respondents were more likely to be research active if they had been awarded a fellowship, an observation that has also been made in medical clinical academic careers [23]. A lack of integration of clinical academic roles across clinical and academic departments and lack of clear career pathways were identified as potential problems. Trusson and colleagues [24] also identified that clear clinical academic career pathways were needed for NMAHPs. Indeed, even the definition of ‘clinical academic’ in non-medical careers may need to be more clearly defined [25]. In the Netherlands, changes in culture, leadership and infrastructure were identified as being required to support clinical academic career pathways in Nursing [26] and effective partnerships between healthcare organisations and universities seem key [27, 28]. All of these issues need to be addressed if NMAHP research capacity and cultureare to be successfully enhanced.

### Strengths and limitations

This was a large study, allowing adequate comparison across professional groups. The online nature of the survey made it accessible to potential respondents and the fixed response nature of the questions (except for the open-ended questions) supported 100% data completeness. The use of the RCC is also a strength, facilitating comparison with the literature. Respondents to our survey may have introduced a potential source of bias and the results may not necessarily be generalisable. For example, respondents were quite mature (64.2% ≥ 40 years old) and therefore the results may not be applicable to traditional notions of ‘early career researcher’. Similarly, less than a third described their role as ‘clinician’ and therefore the results are relevant to a much wider constituency. Although the healthcare organisation response rates were relatively low (N&M 7.2%, AHPs 7.6%), the relative proportions of N&M to AHP respondents (70% [184/264] to 30% [80/264] respectively) was very similar to the proportions employed at the healthcare organisation (71% [2,567/3,618] to 29% [1,051/3,618] respectively), and to National Health Service England workforce data (76% [338,663/446,405] to 24% [107,742/446,405] respectively) (based on October 2021 data, [29]). Such observations may support the validity of the N&M and AHP comparison. The university staff response rate was much higher at 25.4%. Care needs to be taken with interpretation of response rates due to uncertainty about the comparability of the numerators and denominators in the different constituent groups.

It should be noted that there were differences in the proportion of respondents with postgraduate qualifications (N&M 39.5% and AHPs 54.9%). Although there were no group differences in perceived individual success or skills level (Table 4), responses could have been moderated by postgraduate training (e.g. training may theoretically set a higher relative benchmark for judging individual success or skills). Similarly, postgraduate training may have explained some of the differences between N&M and AHPs in the priorities for support and training, with AHPs identifying more specific support needs around statistical analysis, writing for publications and grant applications for example.

It should be acknowledged that a 1–9 scale version of the RCC was used [19] as opposed to the original 1–10 version [13]. Subsequent conversion of scores to a 10-point scale has facilitated comparison with other literature but created median scores that were not whole numbers. Respondents may have used a 10-point scale slightly differently, although the impact on findings is likely to have been minimal.

## Conclusions

Rich information has been generated to develop strategy to enhance NMAHP research capacity and culture. Much of this strategy could be generic but some nuances may be required to address some specific differences between professional groups, particularly related to perceived team success/skills and priorities for support and development. The key learning points to assist with future strategy development have been summarised in Table 7.

**Table 7:** Summary of key findings

• Team success or skill in planning guided by evidence was rated high
• Team success or skill in applying for external funding was rated low
• N&M colleagues rated their team’s success or skill levels more favourably than AHPs
• Individual success or skill in finding and reviewing literature was rated high
• Individual success or skill in applying for external funding was rated low
• The top three barriers were ‘lack of time for research, ‘other work roles take priority’ and ‘lack of suitable backfill’
• The top three motivators were ‘to develop skills’, ‘increased job satisfaction’ and ‘career advancement’
• The top three items for support were ‘Mentorship for my team’, ‘Mentorship for me’ and ‘In-service training with my team’
• The top three topics for learning were ‘Service evaluation’, ‘Funding opportunities’ and ‘Audit’
• Key themes were ‘Employment & staffing’, ‘Professional services support’, ‘Clinical and academic management’, ‘Training & development’, ‘Partnerships’ and ‘Operating principles’

## Supplementary Information

**Additional file 1: Table Additional file 1. **Information aboutdemographics, professional role, and qualifications.

**Additional file 2: Table Additional file 2. **Mean values for theperceived helpfulness of different types of support.

**Additional file 3: Table Additional file 3. **Mean values for theperceived interest in learning more about specific topics.

## Data Availability

The datasets used and/or analysed during the current study are available from the corresponding author on reasonable request.
