# Supplementary material for: Nursing, midwifery, and allied health professions research capacities and cultures: a survey of staff within a university and acute healthcare organisation
Source: BMC Health Serv Res. 2023 Jun 16;23:647. doi: 10.1186/s12913-023-09612-3 (PMC10276387; doi:10.1186/s12913-023-09612-3)
Supplement: Supplementary file 1 — Additional file 1: Table Additional file 1. Information aboutdemographics, professional role, and qualifications. [file 12913_2023_9612_MOESM1_ESM.docx]

**Table, Additional File 1. Information about demographics, professional role, and qualifications.** AHPs = Allied Health Professions, A level = Advanced level, GCSE = General Certificate of Secondary Education, HE = Higher Education, HNC = Higher National Certificate, HND = Higher National Diploma, MRes = Master of Research, MSc = Master of Sciences, N&M = Nursing & Midwifery, O level = Ordinary level, PGCE = Postgraduate Certificate in Education

|  | **N&M**  **(n=223)** | **AHPs**  **(n=133)** | **Other**  **(n=60)** | **Total**  **(n=416)** |
| --- | --- | --- | --- | --- |
| ***“I am completing this questionnaire as a…”*** | | | | |
| Student | 6 (2.7%) | 25 (18.8%) | 16 (26.7%) | **47 (11.3%)** |
| Employee of Coventry University | 33 (14.8%) | 28 (21.1%) | 8 (13.3%) | **69 (16.6%)** |
| Employee of University Hospitals Coventry & Warwickshire NHS Trust | 184 (82.5%) | 80 (60.3%) | 36 (60%) | **300 (72.1%)** |
| ***“How long have you been working in the health industry?”*** | | | | |
| <5 years | 7 (3.2%) | 16 (14.8%) | 8 (18.2%) | **37 (10.0%)** |
| 5-10 years | 42 (19.4%) | 17 (15.7%) | 5 (11.4%) | **55 (14.9%)** |
| >10 years | 168 (77.4%) | 75 (69.4%) | 31 (70.5%) | **277 (75.1%)** |
| ***“What is your age?”*** | | | | |
| <20 | 0 (0.0%) | 5 (3.8%) | 0 (0.0%) | **5 (1.2%)** |
| 20-29 | 11 (4.9%) | 16 (12.0%) | 13 (21.7%) | **40 (9.6%)** |
| 30-39 | 52 (23.3%) | 41 (30.8%) | 11 (18.3%) | **104 (25.0%)** |
| 40-49 | 75 (33.6%) | 41 (30.8%) | 15 (25.0%) | **131 (31.5%)** |
| 50-60 | 74 (33.2%) | 29 (21.8%) | 16 (26.7%) | **119 (28.6%)** |
| >60 | 11 (4.9%) | 1 (0.8%) | 5 (8.3%) | **17 (4.1%)** |
| ***“What is your gender?”*** | | | | |
| Male | 25 (11.2%) | 21 (15.8%) | 20 (33.3%) | **66 (15.9%)** |
| Female | 195 (87.4%) | 109 (82.0%) | 39 (65.0%) | **343 (82.5%)** |
| Prefer not to say | 3 (1.3%) | 2 (1.5%) | 1 (1.7%) | **6 (1.4%)** |
| Other | 0 (0.0%) | 1 (0.8%) | 0 (0.0%) | **1 (0.2%)** |
| ***“My primary role is…”*** | | | | |
| Undergraduate student | 4 (1.8%) | 20 (15.0%) | 5 (8.3%) | **29 (7.0%)** |
| Postgraduate student | 26 (11.7%) | 5 (3.8%) | 12 (20.0%) | **43 (10.3%)** |
| Doctorate student | 1 (0.4%) | 1 (0.8%) | 0 (0.0%) | **2 (0.5%)** |
| Clinician | 56 (25.1%) | 57 (42.9%) | 14 (23.3%) | **127 (30.5%)** |
| Lecturer | 23 (10.3%) | 19 (14.3%) | 1 (1.7%) | **43 (10.3%)** |
| Manager | 36 (16.1%) | 13 (9.8%) | 10 (16.7%) | **59 (14.2%)** |
| Practice educator | 16 (7.2%) | 6 (4.5%) | 1 (1.7%) | **23 (5.5%)** |
| Researcher | 7 (3.1%) | 3 (2.3%) | 7 (11.7%) | **17 (4.1%)** |
| Other | 54 (24.2%) | 9 (6.8%) | 10 (16.7%) | **73 (17.5%)** |
| ***“Are you currently working in an Advanced Clinical Practice role?”*** | | | | |
| Yes | 21/56  (37.5%) | 11/57  (19.3%) | 8/14  (57.1%) | **40/127**  **(31.5%)** |
| ***“Currently, my highest qualification is…”*** | | | | |
| Doctorate | 10 (4.5%) | 14 (10.5%) | 12 (20.0%) | **36 (8.7%)** |
| Other higher degree e.g. MRes, MSc | 55 (24.7%) | 41 (30.8%) | 20 (33.3%) | **116 (27.9%)** |
| PGCE | 2 (0.9%) | 5 (3.8%) | 0 (0.0%) | **7 (1.7%)** |
| Other postgraduate qualification (including professional) | 21 (9.4%) | 13 (9.8%) | 4 (6.7%) | **38 (9.1%)** |
| First degree | 58 (26%) | 28 (21.1%) | 6 (10.0%) | **92 (22.1%)** |
| First Degree with Qualified Teacher Status (QTS) | 1 (0.4%) | 2 (1.5%) | 0 (0.0%) | **3 (0.7%)** |
| Other qualifications at first degree level (including professional) | 19 (8.5%) | 8 (6.0%) | 3 (5.0%) | **30 (7.2%)** |
| Diploma of HE | 34 (15.2%) | 6 (4.5%) | 5 (8.3%) | **45 (10.8%)** |
| HND/HNC | 0 (0.0%) | 2 (1.5%) | 0 (0.0%) | **2 (0.5%)** |
| Other undergraduate qualification (including professional) | 6 (2.7%) | 1 (0.8%) | 4 (6.7%) | **11 (2.6%)** |
| A level, Scottish higher or equivalent | 1 (0.4%) | 6 (4.5%) | 1 (1.7%) | **8 (1.9%)** |
| O level/GCSE or equivalent | 4 (1.8%) | 3 (2.3%) | 2 (3.3%) | **9 (2.2%)** |
| Other qualification | 11 (4.9%) | 3 (2.3%) | 2 (3.3%) | **16 (3.8%)** |
| No qualifications | 0 (0.0%) | 0 (0%) | 0 (0.0%) | **0 (0.0%)** |
| Not known | 1 (0.4%) | 1 (0.8%) | 1 (1.7%) | **3 (0.7%)** |
