# Supplementary material for: Nursing, midwifery, and allied health professions research capacities and cultures: a survey of staff within a university and acute healthcare organisation
Source: BMC Health Serv Res. 2023 Jun 16;23:647. doi: 10.1186/s12913-023-09612-3 (PMC10276387; doi:10.1186/s12913-023-09612-3)
Supplement: Supplementary file 2 — Additional file 2: Table Additional file 2. Mean values for theperceived helpfulness of different types of support. [file 12913_2023_9612_MOESM2_ESM.docx]

**Table, Additional File 2. Mean values for the perceived helpfulness of different types of support.** Answers were scored as follows: Extremely helpful = 4; Very helpful = 3; Moderately helpful = 2; Slightly helpful = 1; Not at all helpful = 0. Mean values in excess of 3 (i.e. ‘very helpful’) have been highlighted in green. Mean values in excess of 2 (i.e. ‘moderately helpful’) have been highlighted in amber. AHPs = Allied Health Professions, CfCE = Centre for Care Excellence, N&M = Nursing & Midwifery, NMAHP = Nursing, Midwifery & Allied Health Professions.

| ***“How helpful do you think the following types of support would be? (select the helpfulness of each)”*** | | | | |
| --- | --- | --- | --- | --- |
|  | **Other**  **(n=60)** | **N&M**  **(n=223)** | **AHPs**  **(n=133)** | **Total**  **(n=416)** |
| Mentorship for my team | [1] 3.27 | [1] 3.22 | [4] 3.20 | **[1] 3.22** |
| Mentorship for me | [5] 3.03 | [2] 3.11 | [7] 3.10 | **[2] 3.10** |
| In-service training with my team | [=3] 3.05 | [3] 3.06 | [8] 3.09 | **[3] 3.07** |
| Support with project grant applications | [=7] 2.98 | [=5] 2.97 | [3] 3.21 | **[4] 3.05** |
| Support with statistical analysis | [=3] 3.05 | [8] 2.89 | [1] 3.29 | **[5] 3.04** |
| Support with study design / methodology | [6] 3.02 | [=5] 2.97 | [6] 3.11 | **[6] 3.02** |
| Support with writing for publication | [10] 2.88 | [7] 2.92 | [2] 3.22 | **[7] 3.01** |
| Research methods workshops | [=7] 2.98 | [4] 2.98 | [9] 3.05 | **[8] 3.00** |
| Support with qualitative data analysis | [2] 3.07 | [9] 2.84 | [5] 3.14 | **[9] 2.97** |
| Support with fellowship applications | [=11] 2.85 | [12] 2.71 | [10] 3.02 | **[10] 2.83** |
| Research priority setting with my team | [=11] 2.85 | [10] 2.74 | [13] 2.95 | **[11] 2.82** |
| Peer review (e.g. abstracts, papers or grant applications) | [=11] 2.85 | [=13] 2.69 | [11] 2.99 | **[=12] 2.81** |
| Networking opportunities with other NMAHP researchers | [9] 2.90 | [=13] 2.69 | [12] 2.96 | **[=12] 2.81** |
| Facilitated action learning sets (small groups working on specific problems) | [16] 2.68 | [11] 2.72 | [14] 2.89 | **[14] 2.77** |
| Advice on clinical academic careers | [17] 2.62 | [15] 2.66 | [15] 2.86 | **[15] 2.72** |
| Regular CfCE seminars | [15] 2.72 | [=16] 2.59 | [16] 2.66 | **[16] 2.63** |
| Annual CfCE conference | [14] 2.77 | [=16] 2.59 | [17] 2.50 | **[17] 2.59** |
| **Mean** | **2.92** | **2.84** | **3.01** | **2.91** |
