# Supplementary material for: Nursing, midwifery, and allied health professions research capacities and cultures: a survey of staff within a university and acute healthcare organisation
Source: BMC Health Serv Res. 2023 Jun 16;23:647. doi: 10.1186/s12913-023-09612-3 (PMC10276387; doi:10.1186/s12913-023-09612-3)
Supplement: Supplementary file 3 — Additional file 3: Table Additional file 3. Mean values for theperceived interest in learning more about specific topics. [file 12913_2023_9612_MOESM3_ESM.docx]

**Table, Additional File 3. Mean values for the perceived interest in learning more about specific topics.** Answers were scored as follows: Extremely interested = 4; Very interested = 3; Moderately interested = 2; Slightly interested = 1; Not at all interested = 0. Mean values in excess of 2 (i.e. ‘moderately interested’) have been highlighted in amber. Mean values in excess of 1 (i.e. ‘slightly interested’) have been highlighted in yellow. AHPs = Allied Health Professions, CU = Coventry University; N&M = Nursing & Midwifery, NMAHP = Nursing, Midwifery & Allied Health Professions, UHCW = University Hospitals Coventry & Warwickshire NHS Trust.

| ***“How interested are you in learning more about the following topics? (select your level of interest for each)”*** | | | | |
| --- | --- | --- | --- | --- |
|  | **Other**  **(n=60)** | **N&M**  **(n=223)** | **AHPs**  **(n=133)** | **Total**  **(n=416)** |
| Service evaluation | [=4] 2.62 | [2] 2.36 | [1] 2.53 | **[1] 2.45** |
| Funding opportunities | [1] 2.72 | [3] 2.32 | [2] 2.44 | **[2] 2.42** |
| Audit | [11] 2.47 | [1] 2.43 | [10] 2.28 | **[3] 2.38** |
| Critical appraisal | [3] 2.67 | [4] 2.30 | [5] 2.35 | **[4] 2.37** |
| Ethics | [2] 2.70 | [5] 2.26 | [=8] 2.31 | **[5] 2.34** |
| Systematic reviews | [8] 2.55 | [6] 2.23 | [13] 2.24 | **[=6] 2.28** |
| Writing for publication | [=6] 2.57 | [9] 2.13 | [3] 2.41 | **[=6] 2.28** |
| Qualitative research design | [16] 2.40 | [8] 2.14 | [=8] 2.31 | **[8] 2.23** |
| Qualitative data analysis | [=13] 2.43 | [=11] 2.07 | [4] 2.39 | **[9] 2.22** |
| NMAHP research at UHCW | [=4] 2.62 | [13] 2.04 | [=6] 2.33 | **[10] 2.21** |
| Statistics | [9] 2.50 | [16] 2.01 | [=6] 2.33 | **[11] 2.19** |
| Quantitative research design | [=13] 2.43 | [=11] 2.07 | [14] 2.23 | **[=12] 2.17** |
| Writing abstracts | [10] 2.48 | [15] 2.02 | [11] 2.27 | **[=12] 2.17** |
| Research governance | [=6] 2.57 | [10] 2.09 | [15] 2.12 | **[14] 2.17** |
| Database searching | [17] 2.30 | [7] 2.17 | [19] 2.03 | **[15] 2.14** |
| Clinical academic careers | [15] 2.42 | [14] 2.03 | [16] 2.10 | **[16] 2.11** |
| Writing grants | [12] 2.45 | [18] 1.84 | [12] 2.26 | **[17] 2.06** |
| NMAHP research at CU | [18] 2.27 | [17] 1.85 | [=17] 2.09 | **[18] 1.99** |
| Academic research careers | [19] 2.20 | [19] 1.80 | [=17] 2.09 | **[19] 1.95** |
| **Mean** | **2.49** | **2.11** | **2.27** | **2.22** |
